# Increasing the Oxygen Consumption in Hermetic Grain Storage Using Grain Weevils (*Sitophilus granarius*)

**DOI:** 10.3390/insects15110845

**Published:** 2024-10-29

**Authors:** Christina Müller-Blenkle, Cornel S. Adler

**Affiliations:** Julius Kühn-Institut, Institute for Ecological Chemistry, Plant Analysis and Stored Product Protection (ÖPV), Königin-Luise-Str. 19, D-14195 Berlin, Germany; cornel.adler@julius-kuehn.de

**Keywords:** hermetic storage, *Sitophilus granarius*, critical oxygen thresholds, oxygen consumption

## Abstract

Loss of stored plant products due to insect infestation is a problem that is likely to increase with global warming. Improved storage under hermetic conditions in oxygen deficiency can prevent or control infestation and preserve product quality. Oxygen levels in hermetic storage decrease due to different factors, one of which is the oxygen consumption of the insects present. Experiments were carried out using varying numbers (25, 50, or 200) of all developmental stages of the grain weevil *Sitophilus granarius* L. (eggs, larvae, pupae, and adult beetles) caged and placed in sealed 30-L containers containing 22 kg of wheat for at least 21 weeks. Oxygen levels were measured at regular intervals. The oxygen consumption depended on the number of insects and went below the critical threshold of 3% for *S. granarius* survival in most of the trials. Some surviving beetles were observed at the end of the hermetic experiments and 12 weeks afterwards during control for progeny, when oxygen levels did not fall below the critical threshold or the low level could not be maintained for a sufficient time. Monitoring oxygen levels in hermetic storage is therefore essential to ensure safe storage over long periods.

## Simple Summary

Insects can destroy stored products such as grains. With warmer temperatures caused by climate change, insects can breed faster and can spoil more grain that is lost for human consumption. In gas-tight (hermetic) storage the oxygen is consumed by the grain and by insects until the oxygen level is too low for any living organism to survive. The experiments described here use different numbers of grain weevils in wire mesh cages to reduce oxygen in gas-tight containers filled with wheat. Low oxygen levels below 3% for several weeks led to the death of the insects. If the period of low oxygen levels was too short, surviving beetles and offspring could be found. The results show, that insects can help to reduce oxygen levels to a low level that is safe for long-term storage. This knowledge could be used to improve hermetic grain storage.

## 1. Introduction

Insects, rodents, and moulds are the cause of losses in stored products all over the world. While the problem is mainly neglected in temperate climates with good food supply, the losses in regions with higher temperatures are significant and add to existing food shortages [1].

With climate change, the harvest volume is expected to decrease due to extreme weather, while losses of stored products can increase due to improved conditions for insects and moulds caused by rising temperatures [2]. Whereas the already present storage species can produce more generations per year, new stored product insects from warmer climates might find better conditions, thus further increasing stored product infestation pressure. Therefore, preventing infestation of stored products is of great importance.

The project AVoiD (Abwehr von Vorratsschädlingen in Deutschland/Preventing stored product pests in Germany) addresses both, the inventory of stored product insects in Germany, including possible population shifts [3], and ways to improve storage conditions by hermetic storage. The focus here is on hermetic storage that is durable, sustainable, easy to use, and affordable for both the European market and small farmers in the Global South. Different types of hermetic storage are compared, and ways for improvement are investigated.

Hermetic (gas-tight) storage on the one hand avoids the attraction of insects towards the stored product since attractive smells cannot leave, e.g., the silo bin. On the other hand, in gas-tight enclosures, a low-oxygen atmosphere develops due to respiration of atmospheric oxygen by grain, microorganisms, and, possibly, insects. Low oxygen levels can maintain grain quality and germination capacity for many years and suppress harmful pests and fungi [4]. Insect populations that were already inside the product when storage commenced cannot develop but succumb due to suffocation.

For hermetic storage, it is important to reduce the oxygen level as quickly as possible since the depletion of oxygen to levels below 3% is the factor that leads to insect mortality rather than an increase of CO_2_ due to respiration to a maximum of some 16% [5]. The increase of CO_2_ in grain under natural conditions, even with high insect infestation, will not reach levels suitable to kill insects [6]. In order to control insects with CO_2_, a minimum of 35% is needed [7]. The amount of oxygen available greatly influences the time necessary for oxygen depletion, and therefore minimizing the air volume in hermetic storage is crucial. Bailey [6] calculated that a headspace of 10% would increase the necessary time by 15%, while it would require 75% more time when the headspace increased to 50%. Besides headspace volume, the rate of oxygen reduction depends on different abiotic factors such as grain moisture and temperature, as well as biotic factors such as the presence of microorganisms and insects [8]. The grain itself consumes oxygen, but to accelerate the process other factors could be added. Insects can considerably add to the consumption of oxygen in hermetic structures. Oxygen consumption of insects has been investigated by different authors showing differences between species but also between developmental stages of the same species (e.g., [9,10,11,12]).

While many storage pests do not survive oxygen levels below 3% for a period of about 6 days at 20 °C, some developmental stages of *S. granarius* are more tolerant toof low oxygen levels [13]. The pupae of this species show the highest tolerance (55 days at 15°, 41 days at 20 °C), followed by the eggs [13]. Annis [14] confirmed that pupae of the genus *Sitophilus* are the most tolerant stage of the most tolerant stored product insect genus. Therefore, conditions that control an infestation of *S. granarius* are also suitable for other species.

The present experiments were carried out to examine the oxygen consumption and the survival of different developmental stages of *S. granarius* placed in hermetic containers with wheat as a means to increase oxygen consumption in hermetic storage.

## 2. Materials and Methods

Ten hermetic 30-L cider containers (Speidel Tank- und Behälterbau GmbH, Ofterdingen, Germany) were used for the experiments, which, in fact, hold 33.35 L plus 0.5 L inside the lid. Therefore, the air volume inside the container was 33.85 L. Containers were filled with 30 L (22 kg) wheat grain. The grain moisture content was measured at the beginning (14.6–14.8%) and at the end (14.5–14.9%) of the hermetic phase of the experiment using an HE 50 (Pfeuffer, Kitzingen, Germany) grain moisture meter determining the electric conductivity.

**Figure 1:**
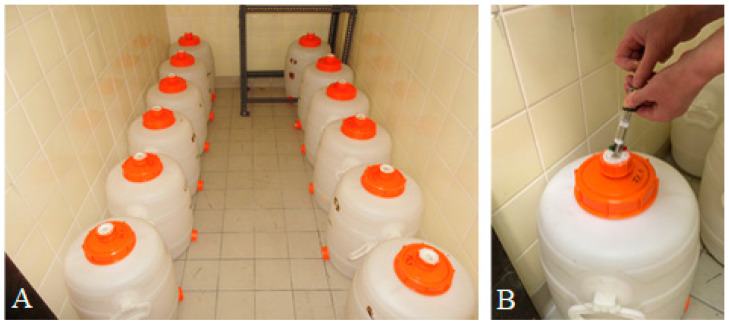
(**A**): Hermetic containers in a climatized chamber. (**B**): Sampling of oxygen using a syringe.

The containers were placed in a rearing chamber with wall heating (Figure 1) in temperatures between 21.5 and 23 °C in the first 15 weeks of the experiments, which then decreased slightly to 20.5 to 21.5 °C in winter, measured using an EL-USB-2 data logger (Lascar Electronics, Whiteparish, UK). The opening of 15 mm diameter at the top of the container was covered with a silicon septum to allow gas sampling with a glass syringe for oxygen measurements using a Toray LF 750 (Lippke Handels GmbH, Neuwied, Germany). For the first seven weeks, oxygen levels were measured regularly, if possible every working day, taking one 10 mL sample from the headspace of each container. Afterwards the measurement interval was extended to about twice a month.

Insect specimens were taken from a weekly breeding batch of *S. granarius*, providing specimens of six defined age groups (see Table 1). For the breeding batch, each week 16 mL of adult beetles of mixed sex were placed on 142 g of wheat were they could lay eggs for three days. Afterwards, the beetles were removed and the grain was stored in a 4.54 m^3^ climate chamber with wall heating at 25 ± 1 °C and 65 ± 5% rh. Temperature was controlled by an Extech Rh520A data logger. Humidity was set by a Venta LW15 humidifier (Venta-Luftwäscher GmbH, Weingarten, Germany), regulated by a humidistat HG mini (Galltec Mess- und Regeltechnik GmbH, Bondorf, Germany).

Experiments were carried out using three different amounts of insects, 25, 50, and 200 specimens per developmental stage. One container was used as a control containing wheat but no insects. Since the larvae are hidden inside the kernels, the numbers of kernels were counted, in the expectation of one larva per kernel. This expectation was supported by the number of emerged adults in the experiments. Therefore, the number of insects at the beginning of the experiments was 6 times approximately 25, 50, or 200, respectively.

**Figure 2:**
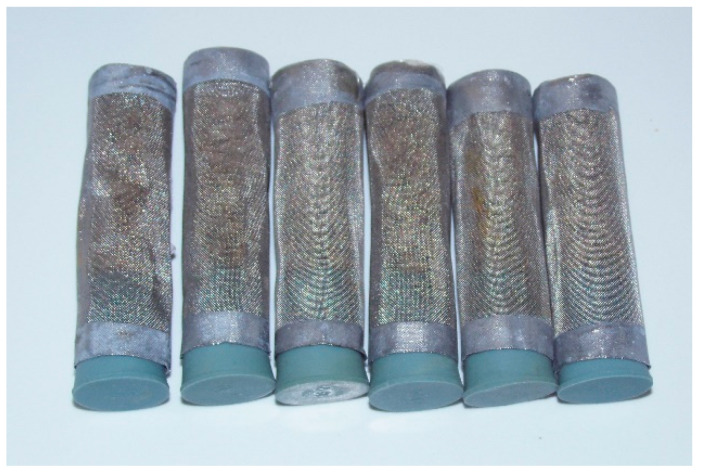
Set of cages for the developmental stages of *S. granarius*.

The kernels of a given developmental stage were counted into wire mesh cages sealed by foam stoppers (Figure 2). Adults were placed into a cage together with one uninfested wheat kernel each. One cage of each developmental stage was placed on a dish made from aluminum foil positioned at the grain surface to prevent soiling of the grain below. Three containers were equipped with the same amount of grain and insects as parallel replicates.

The containers were sealed for about 21 weeks (200 specimens) and 23 weeks (25 and 50 specimens), respectively. The experiments with 25 and 50 specimens started first, followed by the experiment with 200 specimens two weeks later. All experiments ended at the same time since the oxygen levels in the experiment with 200 specimens had already increased above secure level at that time.

At the end of the experiments, the containers were opened, and the content of the cages inspected, placed into a glass jar, and transferred into a climate chamber with weekly control for progeny for another 12 weeks at 25 ± 1 °C and 65 ± 5% rh.

## 3. Results and Discussion

### 3.1. Oxygen Measurements

The decrease of oxygen in the experiments was found to depend on the number of insects. In experiments with 25 insects per stage in only one trial, the level decreased to less than 3% after a period of about 7 weeks. With 50 insects, oxygen fell below this level after 5–6 weeks, except in one case in which the oxygen only dropped below 3% for a day after about 9 weeks. With 200 insects per stage, it took less than 2 weeks for the oxygen to fall below 3%.

In 6 out of 9 trials, oxygen levels of less than 2% could be achieved. The lowest levels of oxygen with 200 insects were reached around 3 weeks after sealing the container. In most cases, the oxygen level increased again, indicating that the amount of oxygen that leaked through the container exceeded the amount that was consumed by insects and grain since the O_2_ concentration results from the depletion of O_2_ and the gas exchange rate with the surroundings [15]. Therefore, the start of the increase is possibly the point where most insects were already dead or inactive. Since all the seals of the containers had been replaced, this leakage might have been due to ageing of the plastic material of the reused containers purchased some 20 years ago.

**Figure 3:**
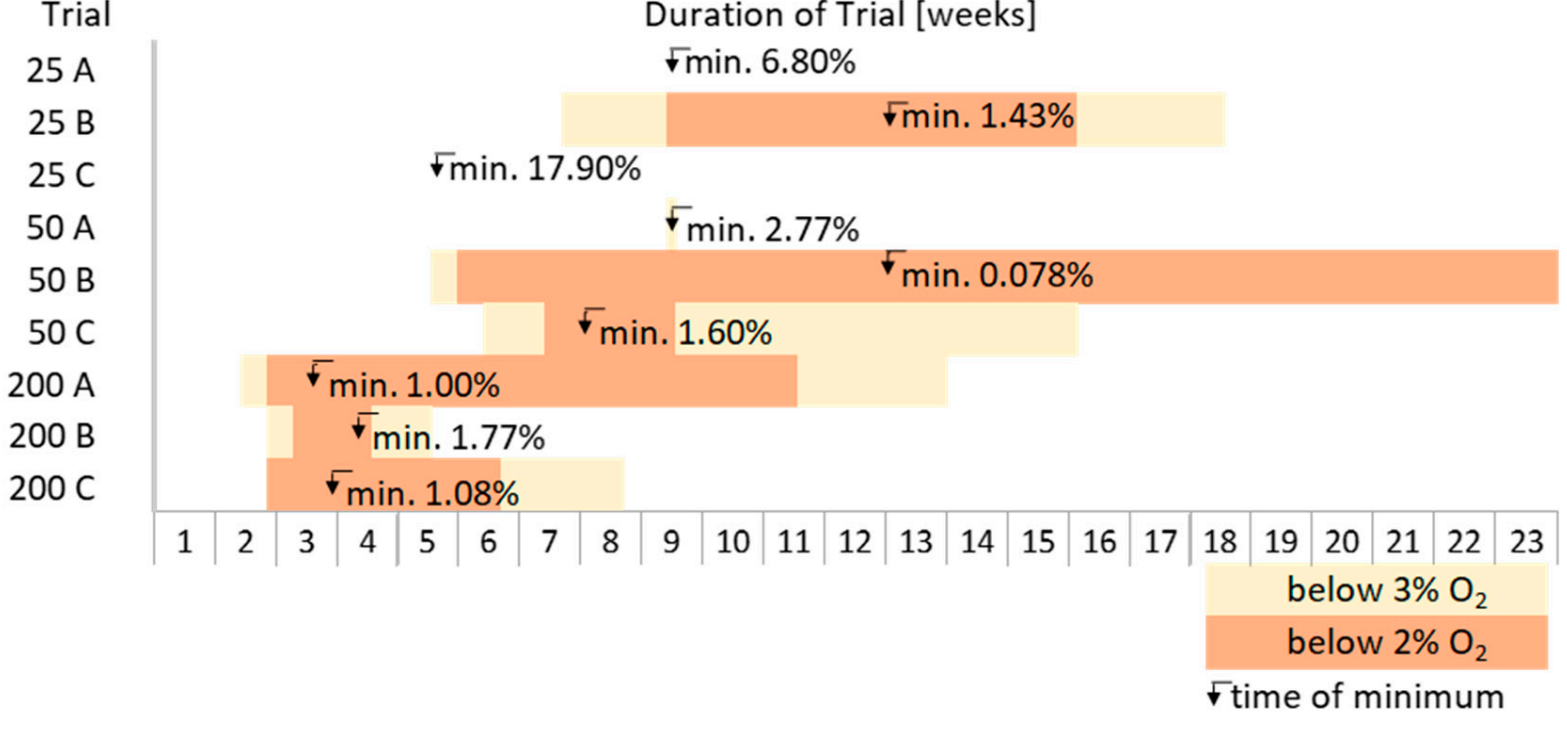
Comparison of oxygen levels during the trials. The trial name indicates the number of insects per stage, followed by A, B, or C for the three replicates. Yellow bars indicate oxygen levels below 3%, and orange bars levels below 2%. The times of the lowest O_2_ levels are marked with arrows, with the measured value next to them.

In seven out of nine trials, the oxygen levels dropped below 3%, although in one case only for one day. In all other cases, an oxygen level below 3% could be achieved for at least 18 days, levels below 2% for at least 8 days but mostly much longer. Figure 3 shows a comparison of periods with low oxygen levels in the different trials.

Bailey [6] obtained comparable results with lightly infested grain containing six *S. granarius* per lb. of grain. The author extrapolated that it would take 39 days for the oxygen to drop to 2%. In the present experiments, we used 22 kg (48.5 lb.) of grain, and therefore the experiments containing 50 insects per stage corresponded to Bailey’s light infestation with oxygen levels going down to 2% after 36 and 46 days.

### 3.2. Development of Insects

**Figure 4:**
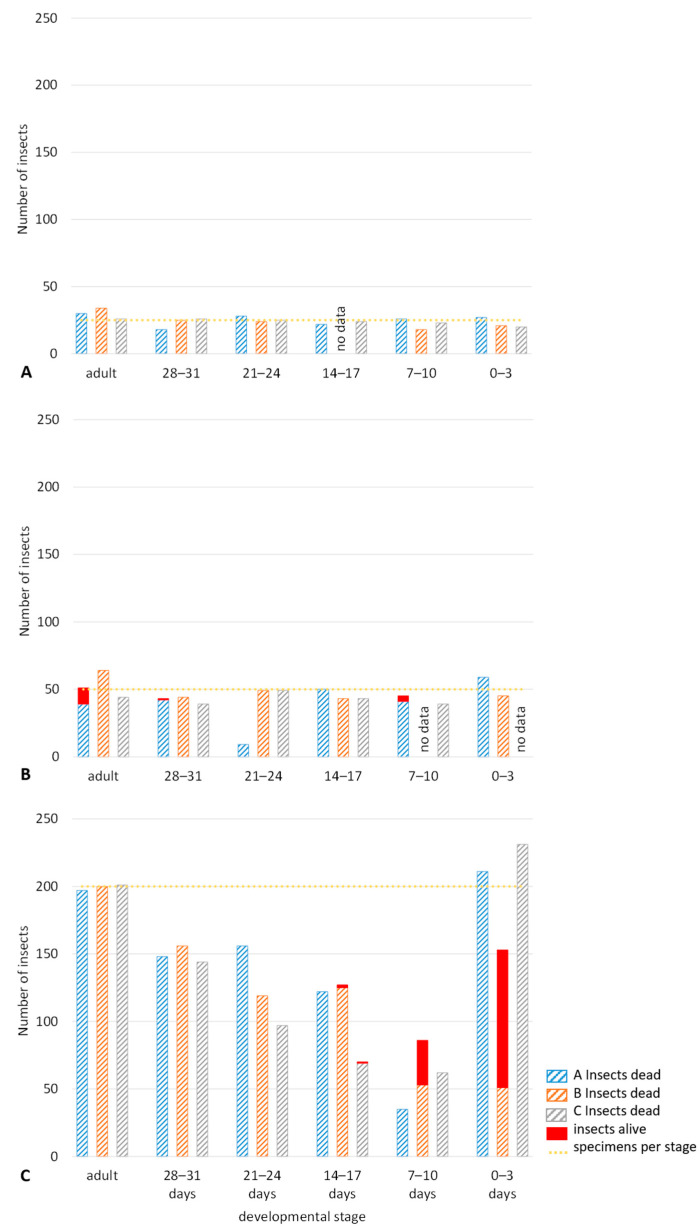
Number of insects present at the end of the experiments, including progeny, over a period of 12 weeks. The three trials are displayed in blue, orange, and grey, with dead insects in hatched columns and live insects marked in red at the top of the column. The orange dotted line indicates the number of specimens introduced at the start of the experiment (**A**): Experiment with 25 specimens of *S. granarius* per age level, (**B**): 50 specimens, and (**C**): 200 specimens. While the number of insects present was more or less the number of introduced specimens in the trials with 25 and 50 insects, the experiment with 200 specimens showed decreasing larval development due to low oxygen levels.

The insect count at the end of the hermetic experiments, including the 12-week control period afterwards, showed that there was limited development of *S. granarius* [16] (Table 2, Figure 4). In only few cases, the number of insects present exceeded the number of introduced insects at the start of the experiment. In the experiments with 200 insects per stage, showing the quickest oxygen reduction, the number of insects present decreased in the different age groups. Only the youngest stage, 0–3 days after oviposition, showed larval development to a certain point before the larvae finally died.

**Figure 5:**
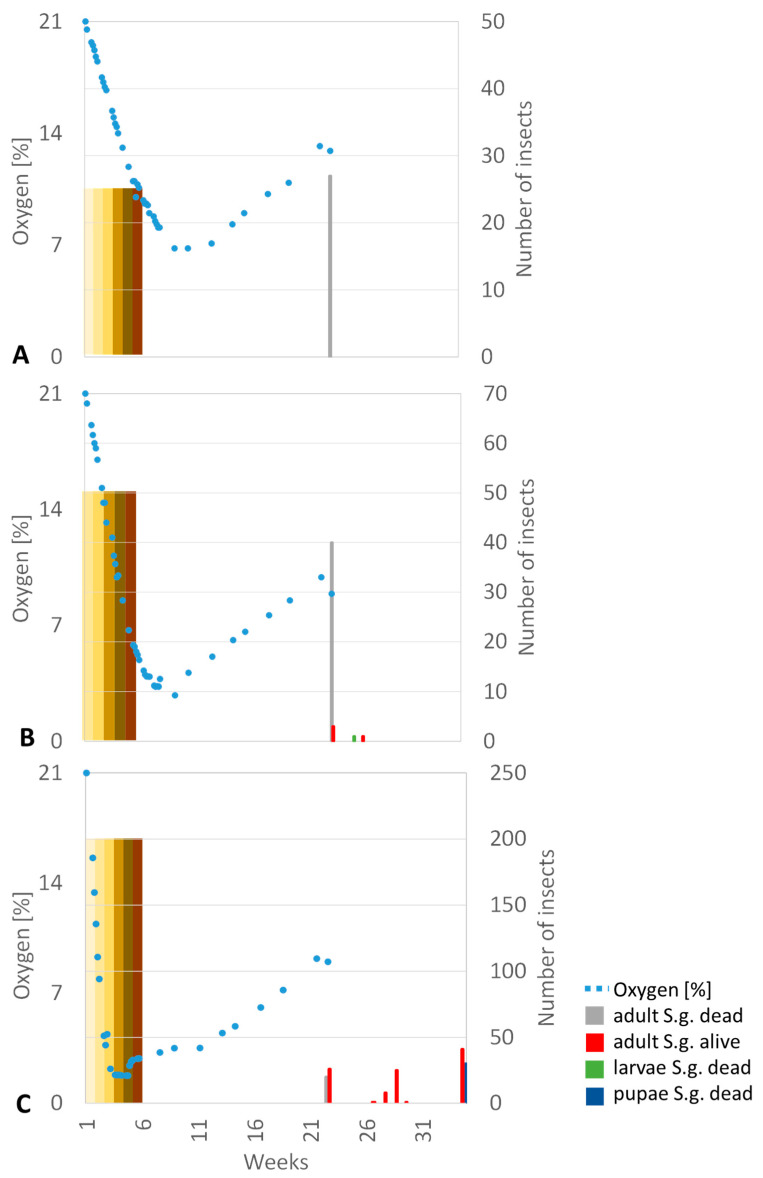
Oxygen level (blue dots) and insect numbers in the three different trials. Adult beetles are marked in grey when dead and red when alive. Dead larvae are marked in green, and dead pupae in dark blue. The colour bar at the left-hand side of the figures indicates the developmental stages during the trials, with light yellow being the very young stage, 0–3 days, leading to dark brown for adults. (**A**): Trial 25 A, eggs 0–3 days, 25 insects per stage. Only dead beetles were found at the end since all the grain had been consumed. (**B**): Trial 50 A, larvae 7–10 days, 50 insects per stage. Most larvae could finish their development, and a few were found alive at the end of the trial and/or as progeny; the food supply was sufficient. (**C**): Trial 200 B, eggs 0–3 days, living beetle were found after the trial with sufficient food supply.

Most larvae used in the trials with 25 individuals per stage were able to complete their development. Therefore, dead beetles were found at the end (for example, Figure 5A). This can be related to the relatively high oxygen levels of 10.6, 6.0, and 18.5% after 6 weeks, which was about the expected development time. Only a few dead larvae or pupae were found. The oxygen content also allowed the beetles’ normal feeding activity, which led to a shortage of grain in the trials. Therefore, further reproduction was impossible, and the beetles died of starvation. This needs to be considered in further experiments.

The oxygen levels in the trials with 50 individuals per stage already decreased quickly in the first weeks. After 6 weeks, only 4.9, 1.9, or 3.5% oxygen was measured. Few survivors and/or progeny could be found in trial 50 A (Figure 5B) where the oxygen level remained below 3% for only one day, but the food supply was also short in this trial. Most larvae were capable of completing their development; only the 0–3 days stage died mainly as larvae or pupae.

200 individuals per stage led to a quick decrease of oxygen, with levels of less than 3% in the first two weeks after sealing. The younger larvae, especially, were exposed to very low levels that went down to 1.0 to 1.77% during their development. Therefore, only a few larvae at the stage of 14–17 days or younger became adults. In most of the trials there was sufficient grain left at the end, which indicates that the insects died early in the experiment. The mortality at this early stage also explains the increase of oxygen already after around 3 to 4 weeks.

Survivors were found in trial 200 B, where the oxygen level increased again to more than 9% at the end of the trial (Figure 5C). The combination of increasing oxygen and sufficient food supply allowed the few beetles that could develop under low oxygen conditions to reproduce successfully.

Njoroge et al. [17] also observed some survival in experiments with rice weevils (*Sitophilus oryzae* L.) after treatment with low oxygen levels. Although no obvious survivors were found after 14 days in 1, 3, or 5% oxygen, beetles emerged during a 45-day post-treatment period from experiments with 3 and 5% O_2_ [17]. Early experiments by Bailey [6] showed a mortality of adult *S. granarius* in oxygen levels of 2.05% after about 17 days, while no mortality was observed at 3.1% oxygen. Reichmuth [13] observed that 1 and 2% oxygen was more effective in controlling *S. granarius* than 0.5 and 3%. The author also pointed out that the metabolism is greater at higher temperatures, and therefore more oxygen is needed; for that, low oxygen levels are more effective at higher temperatures. Pupae and eggs have a low O_2_ consumption, and, consequently, the effect of a deficiency is smaller [13].

Adler and Reichmuth [18] confirmed the tolerance of pupae, followed by eggs, older larvae, younger larvae, and adults. Looking at the present results, survivors were found in the three trials with the shortest periods below 3% oxygen of 1, 18, or 40 days, while no survivors were found in the other trials when the low levels lasted between 67 and 125 days.

In the present experiments, it was the older developmental stages that were exposed to the lowest oxygen levels. Even the youngest stages that were 0–3 days old at the start of the experiment were at least 2–3 weeks (experiments with 200 insects per stage) or at least 5–6 weeks in the other experiments when the oxygen values dropped below 2 or 3%. Since pupae and older larvae are more tolerant of oxygen deficiency, survivors were found mainly in the trials with very young developmental stages at the beginning of the experiment or in those with adults that were able to lay eggs before the oxygen deficiency occurred.

The containers used in the experiments had a volume of nearly 34 L. Of this, 30 L was filled with grain with an estimated intergranular volume of 40% [6], thus containing 12 L of air. Adding the nearly 4 L air in the headspace of the container a volume of about 16 L air would be available for the insects containing some 3.4 L of oxygen.

**Figure 6:**
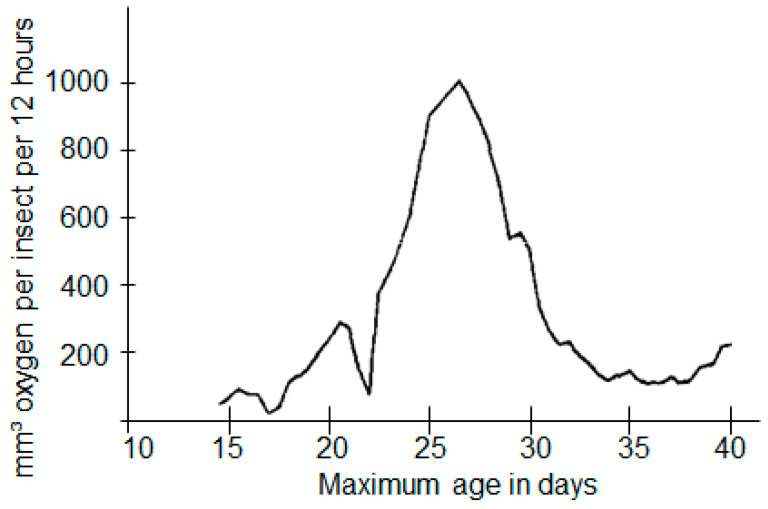
The oxygen consumption pattern of immature *S. granarius* adapted from [11].

**Figure 7:**
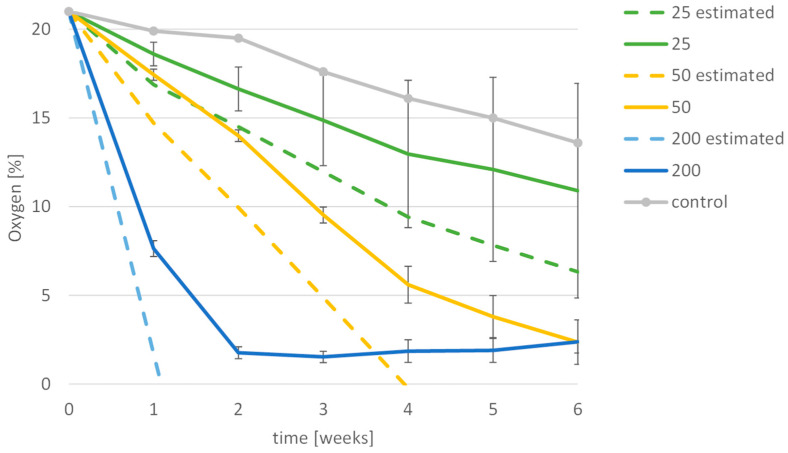
Oxygen consumption in the experiments compared to the data from Bailey [11]. The dashed lines in green (25 insects per stage), yellow (50 insects per stage), and blue (200 insects per stage) show the estimated oxygen consumption based on Bailey [11], while the solid lines of the same colour show the measured data averaged with standard deviation from the three trials for the same amount of insects. The grey line shows the oxygen consumption by grain and microorganisms without insects.

Bailey [11] measured the oxygen consumption of the developmental stages of the grain weevil (*S. granarius*) and showed the highest oxygen consumption was in larvae at an age of about 23 to 30 days, with a maximum consumption of 2000 µL oxygen per day. Following the figure from Bailey (Figure 6) and other results of his [11], the oxygen consumption for each of the developmental stages used was estimated (Table 3). The average consumption per week was multiplied by the number of insects per stage. Figure 7 shows that the present data are comparable to the estimated oxygen consumption based on Bailey’s results.

Although the results are small in scale, they show that adding insects to storage facilities confined to cages or bio-generators could be used to quickly reduce oxygen content under hermetic conditions. Based on the estimations given in Table 3 and the present results, the amount of insects needed on a larger scale was estimated. One ton of wheat contains about 140 L of oxygen in the intergranular space. Using the same composition of insect stages as in the described experiments, about 5400 insects per stage would be necessary to consume the oxygen within 10 days. This would correspond to about 255 g of highly infested wheat per stage (1275 g altogether) and 34 mL of adult beetles kept in insect-safe bags per ton of wheat. To verify the results and to validate the method for larger storage units, experiments using 6 m^3^ hermetic silos containing about 4.5 tons and developmental stages of *S. granarius* are about to start. To address concerns about the introduction of storage pests into storage facilities, further laboratory experiments using species such as *Tenebrio molitor* that are not storage pests or that cannot develop on that particular type of stored product are planned to test for effective and safe oxygen reduction.

Surviving insects can be the basis for new populations since phases of low oxygen levels do not seem to have an effect on fertility [6]. However, Bailey also stated that the oviposition rate was greatly reduced when the oxygen levels were as low as 3% and not suitable for the development of immature stages [6].

The results show that hermetic storage can be successful as disinfestation treatment, and a number of products for hermetic storage are already on the market, which are mainly used for smaller amounts of grain. Nevertheless, it is also obvious that oxygen control is of great importance to detect any leakage and to secure efficacy. In many cases, farmers trust hermetic bags or other structures without checking oxygen levels, which can lead to losses when oxygen leaks in during storage. Infestation can remain at a low level and restart when oxygen levels exceed a certain level, depending on the species involved.

The present and upcoming work is meant to lift hermetic storage to a higher level that is also suitable for large amounts of grain and that avoids large amounts of plastic waste.

## Figures and Tables

**Table 1:** Developmental stages of *S. granarius* [13].

Days after Oviposition	Developmental Stage
0–3	Eggs
7–10	Young Larvae
14–17	Larvae
21–24	Larvae and Pupae
28–31	Pupae
35–38	Adults

**Table 2:** Number of insects present at the end of the experiments, including progeny, over a period of 12 weeks.

Trial	25 A						25 B						25 C					
Max. age [d] *	Ad.	31	24	17	10	3	Ad.	31	24	17	10	3	Ad.	31	24	17	10	3
Adults dead	30	18	28	22	26	27	30	25	22	No data	18	21	24	21	25	19	22	17
Stages dead	0	0	0	0	0	0	4	0	2		0	0	2	5	0	5	1	3
Adults alive	0	0	0	0	0	0	0	0	0		0	0	0	0	0	0	0	0
Stages alive	0	0	0	0	0	0	0	0	0		0	0	0	0	0	0	0	0
Progeny	0	0	0	0	0	0	0	0	0		0	0	0	0	0	0	0	0
Sum	30	18	28	22	26	27	34	25	24		18	21	26	26	25	24	23	20
**Trial**	**50 A**						**50 B**						**50 C**					
Max. age [d] *	Ad.	31	24	17	10	3	Ad.	31	24	17	10	3	Ad.	31	24	17	10	3
Adults dead	37	39	9	50	40	54	40	44	48	42	No data	9	26	37	48	42	38	No data
Stages dead	2	3	0	0	1	5	24	0	1	1		36	18	2	1	1	1	
Adults alive	2	1	0	0	3	0	0	0	0	0		0	0	0	0	0	0	
Stages alive	0	0	0	0	0	0	0	0	0	0		0	0	0	0	0	0	
Progeny	10	0	0	0	1	0	0	0	0	0		0	0	0	0	0	0	
Sum	51	43	9	50	45	59	64	44	49	43		45	44	39	49	43	39	
**Trial**	**200 A**						**200 B**						**200 C**					
Max. age [d] *	Ad.	31	24	17	10	3	Ad.	31	24	17	10	3	Ad.	31	24	17	10	3
Adults dead	197	140	17	0	0	0	200	153	100	60	39	20	201	130	28	1	0	0
Stages dead	0	8	139	122	35	211	0	3	19	65	14	31	0	14	69	68	62	231
Adults alive	0	0	0	0	0	0	0	0	0	2	18	26	0	0	0	1	0	0
Stages alive	0	0	0	0	0	0	0	0	0	0	0	0	0	0	0	0	0	0
Progeny	0	0	0	0	0	0	0	0	0	0	15	76	0	0	0	0	0	0
Sum	197	148	156	122	35	211	200	156	119	127	86	153	201	144	97	70	62	231

* Max. age [d] at start of experiment.

**Table 3:** Estimated weekly average oxygen consumption for different developmental stages of *S. granarius*.

Days after Oviposition	Estimated Oxygen Consumption Based on Figure 6, Averaged for a Week [mm^3^ Oxygen per Insect per 12 h]
0–3	25
7–10	50
14–17	146
21–24	727
28–31	203
35–38	150

## Data Availability

The raw data supporting the conclusions of this article will be made available by the authors on request.
